# Homes on the Riviera

**Published:** 1886-11-06

**Authors:** A. J. Freeman

**Affiliations:** San Remo, Italy


### THE EDITOR'S LETTER-BOX.

[Our Correspondents are reminded that prolixity is a great bar to publication, and that brevity of style and conciseness of statement greatly facilitate early publication.]

### HOMES ON THE RIVIERA.

At the present moment, when so many invalids are anxiously making their winter plans, may I remind the profession, and especially members in the provinces, through your columns of the "Homes for Invalid Ladies of Limited Means," established at Cannes, Mentone, and San Remo? An outlay of between £40 and £50 will cover all expenses, travelling, etc., of a winter in the South at one of these Homes, and there must be many people who would gladly assist an impecunious relative or friend in the restoration to health by this sum.

We shall be glad to forward forms of certificates, etc., at any time for the Home here, or they may be obtained direct by writing to Miss Macdonald Lockhart, The Lee, Lanark. I am, Sir, yours faithfully,

A. J. Freeman, M.D.

San Remo, Italy.

